# RhoGDIβ-induced hypertrophic growth in H9c2 cells is negatively regulated by ZAK

**DOI:** 10.1186/1423-0127-16-11

**Published:** 2009-01-22

**Authors:** Chih-Yang Huang, Li-Chiu Yang, Kuan-Yu Liu, Pao-Hsin Liao, Janet Ing-Yuh Chou, Ming-Yung Chou, Wei-Wen Lin, Jaw-Ji Yang

**Affiliations:** 1 Graduate Institute of Chinese Medical Science, China Medical University, Taichung 404, Taiwan; 2 Graduate Institute of Basic Medical Science, China Medical University, Taichung 404, Taiwan; 3 Department of Health and Nutrition Biotechnology, Asia University, Taichung 413, Taiwan; 4 School of Dentistry, Chung-Shan Medical University Hospital, Taichung 402, Taiwan; 5 Institute of Medicine, Chung-Shan Medical University Hospital, Taichung 402, Taiwan; 6 Chung-Shan Medical University Hospital, Taichung 402, Taiwan; 7 Midwestern University Chicago College of Osteopathic Medicine, Chicago, USA; 8 Cardiovascular Center, Veterans General Hospital, Taichung 407, Taiwan

## Abstract

We found that overexpression of RhoGDIβ, a Rho GDP dissociation inhibitor, induced hypertrophic growth and suppressed cell cycle progression in a cultured cardiomyoblast cell line. Knockdown of RhoGDIβ expression by RNA interference blocked hypertrophic growth. We further demonstrated that RhoGDIβ physically interacts with ZAK and is phosphorylated by ZAK *in vitro*, and this phosphorylation negatively regulates RhoGDIβ functions. Moreover, the ZAK-RhoGDIβ interaction may maintain ZAK in an inactive hypophosphorylated form. These two proteins could negatively regulate one another such that ZAK suppresses RhoGDIβ functions through phosphorylation and RhoGDIβ counteracts the effects of ZAK by physical interaction. Knockdown of ZAK expression in ZAK- and RhoGDIβ-expressing cells by ZAK-specific RNA interference restored the full functions of RhoGDIβ.

## Background

ZAK belongs to the mixed lineage kinases, a family of serine/threonine kinases that are classified as MAP3Ks and can activate the JNK and nuclear factor κB (NFκB) pathway [1]. ZAK induces JNK activation through a dual phosphorylation kinase, JNKK2/MKK7 [2]. To identify effectors of ZAK and to study the ZAK signaling cascade, we used a yeast two-hybrid system to isolate ZAK effectors from a human heart cDNA library. One of the isolated cDNAs encoded Rho GDP dissociation inhibitor beta (RhoGDIβ). RhoGDIβ, also known as Ly-GDI or D4-GDI, belongs to a family of Rho GDP dissociation inhibitors [3-5] that includes RhoGDIα, RhoGDIβ, and RhoGDIγ [6,7] and is thought to regulate the activity and localization of Rho family proteins [8-11]; however, only their ability to sequester RhoGTPases is well understood. RhoGDIs regulate RhoGTPase activity by inhibiting GDP dissociation, maintaining RhoGTPases in an inactive state. RhoGDIα is expressed in all cell types and appears to function as a molecular chaperone for GTPases, shuttling them between the cytosol and the plasma membrane [12]. Therefore, the question remains as to whether RhoGDIβ is actually a RhoGTPase regulator. A recent study indicated that stimulation of T lymphocytes and myelomonocytic cells with phorbol esters leads to RhoGDIβ phosphorylation on serine/threonine residues [13], raising the question of whether RhoGDIβ is involved in a signal transduction pathway in these cells. Thus, details of the regulatory roles of RhoGDIβ remain to be elucidated.

In the present study, we provide evidence that ZAK serves as a RhoGDIβ kinase, and demonstrate the phosphorylation of RhoGDIβ by ZAK *in vitro*, as well as the physical association between ZAK and RhoGDIβ. We further show that this phosphorylation negatively regulates RhoGDIβ-induced hypertrophic growth. Therefore, we speculate that the ZAK-RhoGDIβ interaction maintains ZAK in a hypophosphorylated inactive form.

## Materials and methods

### Immunoprecipitation and detection of ZAK kinase activity

293T cells were transfected with the ZAK expression vector, and cell lysates were prepared in IP buffer (40 mM Tris-HCl, pH 7.5, 1% (w/v) Nonidet P-40 (NP40), 150 mM NaCl, 5 mM EGTA, 1 mM dithiothreitol (DTT), 1 mM phenylmethylsulfonyl (PMSF), 20 mM NaF, proteinase inhibitors (1 mg/ml Leupeptin, 1 mg/ml Pepstatin A, 2.5 mg/ml Aprotinin) (Sigma), and 1 mM sodium vanadate). Cell extracts (200 μg) were incubated with 5 μl of anti-ZAK polyclonal antibody generated in our laboratory for 6 h at 4°C, mixed with 20 μl of protein-A Sepharose suspension, and incubated for an additional hour. Immunoprecipitates were collected by centrifugation and washed three times with IP buffer and twice with kinase buffer (20 mM Tris-HCl, pH7.4, 4 mM MgCl_2_) alone. Protein kinase assays were carried out using a GST-RhoGDIβ fusion protein as the substrate. The GST-RhoGDIβ fusion protein was incubated for 15 min at 25°C with immunoprecipitated ZAK in the presence of kinase buffer (20 mM HEPES, pH 7.6, 1 mM EGTA, 1 mM (DTT), 2 mM MgCl_2_, 2 mM MnCl_2_, 5 mM NaF, 1 mM Na_3_VO_4_, 50 mM NaCl) and [γ-^32^P]ATP. Following incubation, the phosphorylated GST-RhoGDIβ was boiled in SDS sample buffer, and the proteins were subjected to SDS-PAGE on a 15% gel. The gel was dried, and phosphorylated RhoGDIβ substrate was visualized by autoradiography.

### Pull-down assay

293T cells were transfected with the pEGFPC1 ZAK mutant constructs described above. Cell lysates were pre-cleared with glutathione-Sepharose beads, followed by the addition of glutathione-Sepharose beads bound to GST-RhoGDIβ and incubation with rotation for at least 2 h at 4°C in pull-down (PD) buffer (50 mM Tris-HCl, pH 8.0, 150 mM NaCl, 0.5% NP40, 1 mM DTT, 1 mM PMSF, and protease inhibitor mixture). The beads were washed five times with PD buffer and boiled for 5 min in 30 μl of 5× concentrated Laemmli buffer. The bound proteins were then resolved by electrophoresis on 10% SDS-PAGE gels, followed by immunoblotting.

### SiRNA knockdown

We used the expression vector pCDNA-HU6, a derivative of pCDNA3.1/Myc-His(-) with a human U6 promoter, for expression of short hairpin RNAs (shRNAs). Two oligonucleotides, shRNA-F (36 nucleotides) and shRNA-R (41 nucleotides), were synthesized, each of which consisted of a 19-nucleotide stem sequence. The oligonucleotides used for shRNA were as follows: ZAK460iF (GATCCGCCTCTCGGTTCCATAACCATTTCAAGAGAA), ZAK460iR (AGCTTAAAAAGCCTCTCGGTTCCATAACCATTCTCTTGAAA), ZAK1712iF (GATCCGCCAGTGGTTAGATACTCTGATTCAAGAGAT), ZAK1712iR (AGCTTAAAAAGCCAGTGGTTAGATACTCTGATCTCTTGAAT), RhoGDIβ353iF (GATCCGGGATATTGTGTCAGGCCTGATTCAAGAGAT), RhoGDIβ353iR (AGCTTAAAAAGGGATATTGTGTCAGGCCTGATCTCTTGAAT), RhoGDIβ568iF (GATCCGCTGGGAGTGGAACCTGTCGATTCAAGAGAT), and RhoGDIβ568iR (AGCTTAAAAAGCTGGGAGTGGAACCTGTCGATCTCTTGAAT). Stable RhoGDIβ -or ZAK-expressing H9c2 cell lines were transfected with these pCDNA-HU6-shRNA plasmids and a pBABE-puro plasmid as a selection marker. Puromycin-resistant clones were further tested for expression of ZAK or RhoGDIβ after addition of doxycycline to induce expression.

### Cell staining and measurement and growth

H9c2 cells were fixed and permeabilized. Actin filaments were visualized using rhodamine-labeled phalloidin. Cells were examined and photographed using a Zesis Axioskop and a confocol microscope. The cell size was analyzed using Image-Pro Plus software.

To determine cell growth, cells were grown in plated on 6-well dish (5 × 10^3^/well) with the presence of doxycycline to induce the ectopic genes. Cells were count every 24 hours by hemacytometer.

## Results

### Isolation of a ZAK-interacting protein, RhoGDIβ

A fusion protein containing the GAL4 DNA binding domain and ZAK was used as bait in a yeast two-hybrid screen for proteins that associate with ZAK from a human heart cDNA library, as ZAK is highly expressed in heart tissue. Eighty-one clones that specifically interacted with the bait were identified. Restriction enzyme and nucleotide sequence analysis revealed that two of these clones contained a 1.3-kb cDNA with a 127-amino acid open reading frame. The cDNA was amplified by RT-PCR and found to encode the 204-residue human protein RhoGDIβ.

### Interaction between ZAK and RhoGDIβ

**Figure 1:**
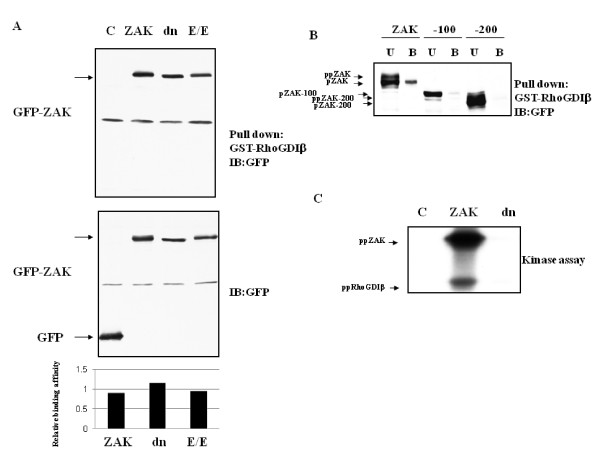
**Differential binding of ZAK to RhoGDIβ and phosphorylation by ZAK**. 293T cells were transiently transfected with the indicated GFP-tagged wild-type or mutant ZAK. After 48 h, cell lysates were harvested and 300 μg of total lysate was used in a pull-down assay with glutathione-Sepharose bead-bound GST-RhoGDIβ. (A) Wild-type ZAK (ZAK), dominant-negative (dn) ZAK, or constitutively active (E/E) ZAK. Bottom panel shows the binding affinity of RhoGDIβ to different forms of ZAK. The relative binding affinity was calculated by the amount of bound form divided by the total loading of RhoGDIβ. (B) Binding of RhoGDIβ to ZAK may maintain ZAK in a hypophosphorylated, inactive state. Unbound (U) ZAK in cell lysates consisted of both hyperphosphorylated (ppZAK) and hypophosphorylated (pZAK) forms. The hypophosphorylated form of ZAK was pulled down by GST-RhoGDIβ. (C) ZAK induces autophosphorylation and phosphorylation of GST-RhoGDIβ. Cell lysates from 293T cells transfected with pEGFPC1 empty vector (C), wild-type ZAK (ZAK), or dominant-negative ZAK (dn) were immunoprecipitated with anti-GFP. The immunopurified ZAK or ZAKdn was incubated with GST-RhoGDIβ in the presence of [γ-^32^P]ATP. Data shown are representative of three independent experiments.

To test whether ZAK and the RhoGDIβ identified by the yeast two-hybrid screen interact *in vitro*, we performed pull-down assays with green fluorescent protein (GFP)-tagged ZAK and glutathione *S*-transferase (GST)-tagged RhoGDIβ. We observed that GST-RhoGDIβ interacted with ZAK, a dominant-negative (dn) ZAK, and a constitutively active (E/E) ZAK (Fig. 1A). We also demonstrate that RhoGDIβ binding to ZAK may be mediated through a region encompassing residues 600–700 (Additional file 1) of ZAK. Furthermore, the fragment corresponding to this region of amino acids 600–700 in ZAK (ZAK 6–7) was pulled down with GST-RhoGDIβ (Additional file 2), suggesting that the binding site for RhoGDIβ is between residues 600–700. Taken together, these data indicate that ZAK physically interacts with RhoGDIβ **t**hrough the region encompassing residues 600–700, and that this interaction is independent of the kinase activity of ZAK, as the kinase-dead mutant (ZAKdn) was pulled down by GST-RhoGDIβ. We also demonstrated that the relative binding affinity of RhoGDIβ to ZAKdn is higher than wild-type ZAK.

We have previously shown that ZAK forms homodimers via its intrinsic leucine zipper domain in order to activate the JNK/SAPK pathway [2]. Upon dimerization, ZAK phosphorylates its dimerized partner, resulting in full activation of ZAK. The data presented here (Fig. 1B) and previously indicate that overexpression of ZAK in cells results in a shift in its mobility by SDS-PAGE as a result of autophosphorylation [2]. Because ZAK might exist in both unphosphorylated and hyperphosphorylated forms *in vivo*, we tested the ability of unphosphorylated and hyperphosphorylated ZAK to bind RhoGDIβ. When unbound ZAK was visualized by immunoblotting, both the unphosphorylated and hyperphosphorylated forms were detected, but only unphosphorylated ZAK was pulled down by GST-RhoGDIβ, where -100 and -200 were the positive and negative binding control, respectively (Fig. 1B). Thus, our result suggests that RhoGDIβ has a higher affinity for unphosphorylated ZAK.

Given the observed physical interaction of RhoGDIβ with ZAK, RhoGDIβ may be a kinase substrate for ZAK. We therefore investigated the potential for RhoGDIβ to be phosphorylated in the presence of ZAK. An *in vitro *kinase assay demonstrated that wild-type ZAK could phosphorylate RhoGDIβ, but the dominant-negative ZAK could not. Moreover, autophosphorylation of ZAK was also detected in this assay (Fig. 1C). Our results demonstrate that RhoGDIβ is able to both bind to and be phosphorylated by ZAK, suggesting that the function of RhoGDIβ may be regulated by ZAK.

### RhoGDIβ induces hypertrophic growth in H9c2 cells

**Figure 2:**
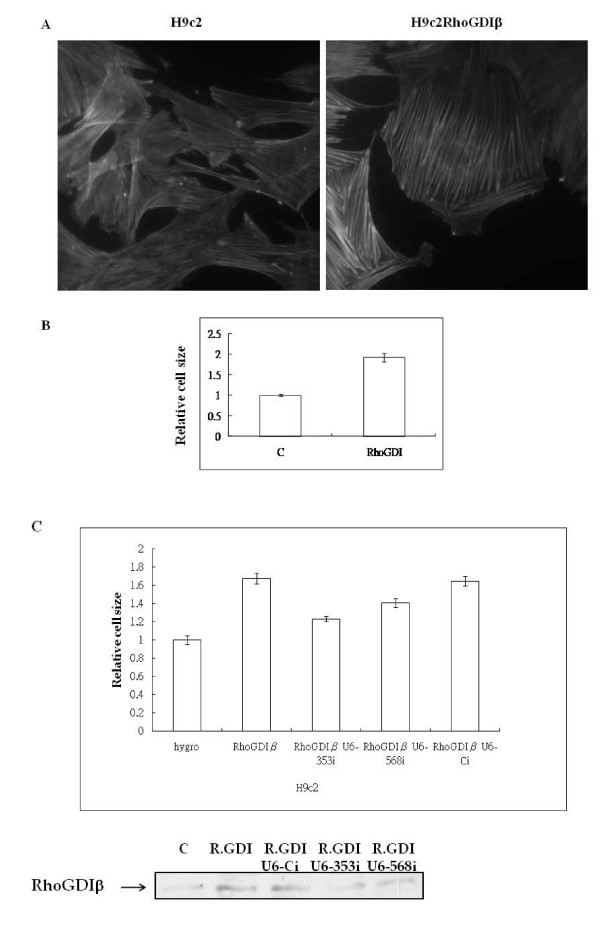
**RhoGDIβ expression in H9c2 cells induces hypertrophic growth**. (A) H9c2 cells stably transfected with empty vector (pTet) or pTet-RhoGDIβ were grown in 10% fetal bovine serum with doxycycline for 3 days. The transfected H9c2 cells were fixed and immunostained with rhodamine-conjugated phalloidin. (B) Relative cell size was measured after 3 days of growth of the transfected H9c2 cells in the presence of doxycycline. (C) Quantification of the cell size of H9c2 RhoGDIβ-expressing cells that also expressed RhoGDIβ siRNA (U6-353i or U6-568i) or scrambled siRNA (U6-Ci).

To characterize the biological effects of RhoGDIβ on cardiac cells, we generated stable H9c2 cardiac cells that expressed RhoGDIβ under tetracycline-responsive transactivator control. In this system, the addition of the tetracycline analog doxycycline induces the expression of RhoGDIβ. Expression of RhoGDIβ resulted in a marked increase in actin fiber organization (Fig. 2A), and cell size increased by approximately 2-fold compared to control cells (Fig. 2B).

### Knockdown of RhoGDIβ restores cell size in RhoGDIβ-expressing H9c2 cells

To confirm the role of RhoGDIβ in hypertrophic growth of RhoGDIβ-expressing H9c2 cells, we knocked down RhoGDIβ expression in these cells by vector-based stable transfection of small interfering RNAs (siRNAs) specific for RhoGDIβ. Two target sequences were used, corresponding to nucleotides 353 to 373 (U6-353i) and 568 to 588 (U6-568i). The siRNA-mediated suppression of RhoGDIβ expression was stable for 60 days (data not shown). RhoGDIβ knockdown cells were smaller than RhoGDIβ-overexpressing cells (Fig. 2C) and had less organized actin fibers (data not shown). These results demonstrate that RhoGDIβ is able to modulate hypertrophic growth in H9c2 cells, and that specific knockdown of RhoGDIβ decreases the enlarged cell size induced by RhoGDIβ.

**Figure 3:**
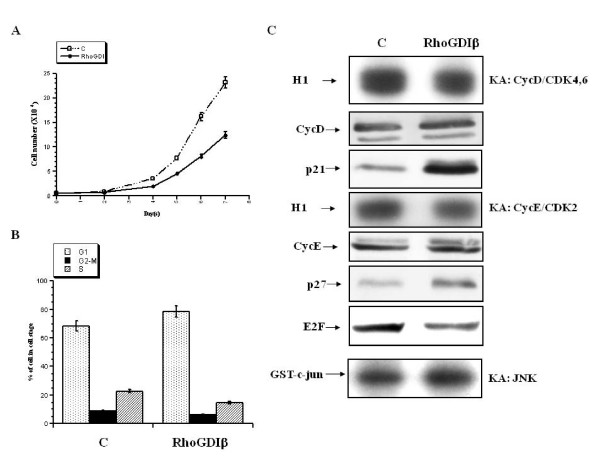
**RhoGDIβ expression in H9c2 cells retards cell cycle progression**. (A) Growth rate of H9c2 cells stably transfected with pTet or pTet-RhoGDIβ grown in 10% fetal bovine serum containing doxycycline. (B) Analysis of RhoGDIβ-expressing cells by flow cytometry. RhoGDIβ-expressing H9c2 cells were grown in 10% fetal bovine serum with doxycycline for 3 days, and cells were stained with propidium iodide and subjected to flow cytometry. The percentages of cells in the different cell cycle phases were calculated using ModFit software. Experiments were performed three times and gave similar results. (C) Expression and activities of various regulatory cell cycle proteins in RhoGDIβ-expressing cells. Total cell extract was analyzed by western blotting and probing with the specific antibodies indicated. Cyclin D or E was immunoprecipitated from RhoGDIβ-expressing cells and analyzed for associated histone H1 kinase activity. JNK activity was analyzed by immunoprecipitating JNK and assessing its activity using GST-c-jun as a substrate.

To assess whether the expression of RhoGDIβ in H9c2 cardiac cells could influence cell cycle progression, we first set out to determine the growth rate of RhoGDIβ-expressing cells compared to parental cells. A total of 5000 cells ectopically expressing RhoGDIβ were cultured in 6-well dishes. The RhoGDIβ-expressing cells grew slower than the parental cells (Fig. 3A). To determine whether the slower growth of the RhoGDIβ-expressing cells was due to blocking of the cell cycle, the cell cultures were trypsinized and their cell cycle distributions were analyzed by flow cytometry. RhoGDIβ-expressing cells had a higher percentage of cells in G_1 _(78.77%) compared to control cells (68.50%). Moreover, fewer RhoGDIβ-expressing cells were in the G_2_/M and S phases (6.35% and 8.71%, respectively) compared to parental cells (14.88% and 22.78%, respectively) (Fig. 3B). These results suggest that RhoGDIβ or its regulated signaling pathways might be involved in regulation of G_1 _checkpoint control.

### Effects of RhoGDIβ on expression of cell cycle regulatory proteins

The expression levels of G_1 _cyclins (cyclin D and E) are rate-limiting for cell cycle progression [14]. However, by western blot analysis, neither cyclin D nor E levels were changed in the low proliferative capacity RhoGDIβ-expressing cells (Fig. 3C). Because cell cycle progression commences in G_1 _with assembly and activation of cyclin D-CDK4/6, followed by the subsequent activation of cyclin E-CDK2 in the G_1_/S transition, we also studied the effect of RhoGDIβ on G_1 _cell cycle regulatory proteins. RhoGDIβ expression also had no effect on the activity of the cyclin D-CDK4/6 complex in H9c2 cells; however, the kinase activity of cyclin E-CDK2 complexes decreased in the RhoGDIβ-expressing cells (Fig. 3C). Because the activities of these CDK complexes can be regulated by the cyclin-dependent kinase inhibitors (CKIs) p21^Waf1/Cip1 ^and p27^Kip1^, we measured their expression levels. We determined that the protein levels of both p21^Waf1/Cip1 ^and p27^Kip1 ^were increased in the RhoGDIβ-expressing cells (Fig. 3C). However, the high p21^Waf1/Cip1 ^expression levels did not decrease the activity of the cyclin D-CDK4/6 complex in these cells. Studies by other investigators have indicated that elevated CKI expression levels are similarly associated with vascular smooth muscle [15] and mesangial cell hypertrophy [16]. Our observations suggest that the level of p21^Waf1/Cip1^, which is regulated by RhoGDIβ or its signaling pathways, might play a role in the response to hypertrophic stimuli. This role, however, appears to be dissociated from the regulation of cyclin D-CDK4/6 activity by p21. Furthermore, levels of p27^Kip1 ^were elevated in RhoGDIβ-expressing cells, and expression was inversely correlated to the kinase activity of cyclin E-CDK2 (Fig. 3C). These results suggest that expression of p27^Kip1 ^might be associated with cell proliferation.

### RhoGDIβ-induced hypertrophic growth is regulated by ZAK

**Figure 4:**
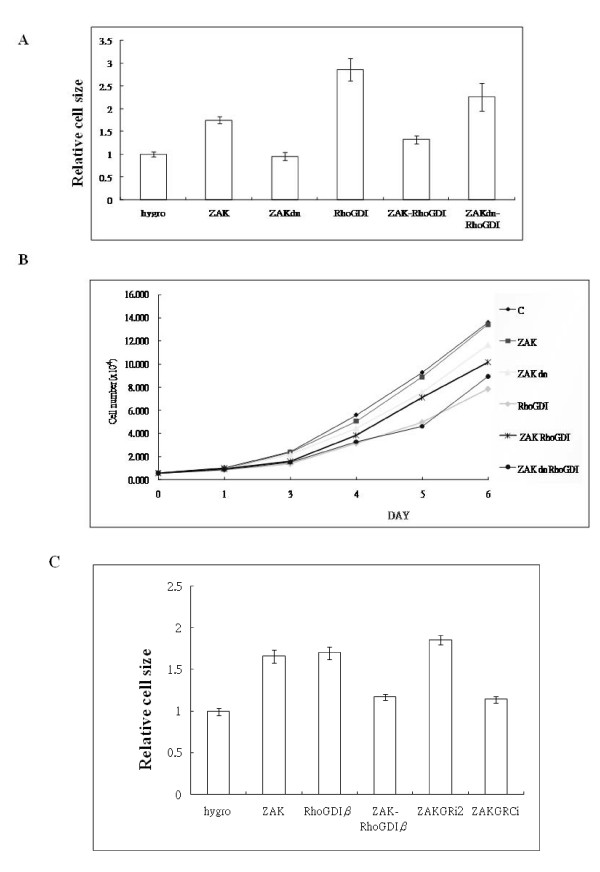
**ZAK reverses the effects of RhoGDIβ induction of hypertrophic growth, cell migration, and suppression of cell cycle progression in H9c2 cells**. (A) H9c2 cells or H9c2 cells that ectopically expressed ZAK, dominant-negative ZAK (ZAKdn), RhoGDIβ, ZAK and RhoGDIβ, or ZAKdn and RhoGDIβ under the control of a bi-directional tetracycline response element were grown in 10% fetal bovine serum with doxycycline for 3 days, and cell size was determined. (B) The growth rate of the same cells shown in (A) grown in 10% fetal bovine serum with doxycycline. (C) SiRNA knockdown of ZAK restores the hypertrophic growth induced by RhoGDIβ in ZAK- and RhoGDIβ-expressing cells (ZAKGRi2).

Given our finding that RhoGDIβ proteins can associate with and be phosphorylated by ZAK, we hypothesized that the function of RhoGDIβ is regulated by ZAK. We used a bi-directional tetracycline-responsive transactivator vector to regulate the expression of RhoGDIβ or ZAK (wt or dn), or both RhoGDIβ and ZAK (wt or dn), in H9c2 cells, and then cell sizes were measured. Consistent with previous studies [17,18], ZAK-expressing or RhoGDIβ-expressing cells showed an increase in cell size of approximately 1.7- or 2.5-fold, respectively (Fig. 4A), and also underwent actin fiber reorganization (data not shown). The co-expression of both ZAK and RhoGDIβ in H9c2 cells resulted in a decrease in cell size from 2.5-fold to 1.2-fold relative to the parental cells. ZAKdn-RhoGDIβ cells did not have a substantially decreased cell size relative to RhoGDIβ-expressing cells (Fig. 4A). These results suggest that the kinase activity of ZAK might play a negative regulatory role in the hypertrophic growth functions of RhoGDIβ in cardiac cells.

### Overexpression of both RhoGDIβ and ZAK reverses RhoGDIβ-regulated cell arrest

As the expression of RhoGDIβ induced G1 arrest in H9c2 cells, but expression of ZAK had no effect on cell cycle progression and ZAK was able to regulate RhoGDIβ functions, we next studied the effects of ZAK on RhoGDIβ's regulation of cell growth rate by examining the growth rate in ZAK- and RhoGDIβ-expressing cells compared with RhoGDIβ-expressing cells. Cells expressing both ZAK and RhoGDIβ grew substantially faster than RhoGDIβ-expressing cells (Fig. 4B). Moreover, ZAKdn- and RhoGDIβ-expressing cells demonstrated the same growth rate as RhoGDIβ-expressing cells.

To determine the role of ZAK in RhoGDIβ-induced hypertrophic growth in H9c2 cells, we compared the cell sizes of ZAK siRNA-, ZAK- and RhoGDIβ-expressing cells (ZAKGRi2), scrambled ZAK siRNA-, ZAK- and RhoGDIβ-expressing cells (ZAKGRCi), and ZAK- and RhoGDIβ-expressing cells. The expression of ZAK siRNA, but not scrambled ZAK siRNA, in cells overexpressing ZAK and RhoGDIβ increased cell size to the same degree as observed for RhoGDIβ-expressing cells (Fig. 4C).

## Discussion

We identified RhoGDIβ as a ZAK-interacting protein in a yeast two-hybrid screen. This led us to investigate whether and how they might regulate one another's functions. RhoGDIα was first identified in bovine brain cytoplasm on the basis of its ability to inhibit GDP dissociation from RhoGTPases. RhoGDIβ, the second GDI identified, shares 67% amino acid identity with RhoGDIα and is almost exclusively expressed in hematopoietic lineages [8]. In contrast to RhoGDIα, RhoGDIβ might bind to and inhibit GDP dissociation from purified RhoGTPases *in vitro*, and appears to be at least 10-fold less potent than RhoGDIα [19]. Moreover, it has been reported that RhoGDIβ can be phosphorylated on tyrosine [20]. Therefore, questions arise with regard to whether RhoGDIβ is an actual regulator of cellular RhoGTPases, its role in the signaling pathway, and its regulation. Upon introduction of RhoGDIβ into rat cardiac H9c2 cells, the cells exhibited hypertrophic growth and a retarded cell cycle. We also demonstrated that RhoGDIβ is phosphorylated by ZAK *in vitro*. It is striking that the co-expression of ZAK and RhoGDIβ in H9c2 cardiac cells resulted in inhibition of the biological functions of RhoGDIβ, indicating that not only does RhoGDIβ potentially physically interact with ZAK, but it may also be negatively regulated by ZAK via phosphorylation. Several studies have also demonstrated that RhoGDIβ is cleaved at its N terminus during apoptosis in a caspase-dependent manner, and that the cleaved RhoGDIβ is retained in the nuclear compartment [21]. This suggests that RhoGDIβ could function in the nucleus.

Numerous studies have implicated RhoGDIβ phosphorylation/dephosphorylation in the regulation of RhoGTPases/RhoGDIβ complex dissociation and mediation of RhoGTPase activation [22,23]. We found that RhoGDIβ was able to associate with a mixed lineage kinase, ZAK, resulting in phosphorylation of RhoGDIβ. We are currently mapping the phosphorylation sites of RhoGDIβ. To further study the role of ZAK in regulating the activity of RhoGDIβ, we used a bi-directional tet-on inducible system to express both ZAK and RhoGDIβ in H9c2 cardiac cells. Our results demonstrate that the hypertrophic growth phenotype was inhibited by coexpression of ZAK and RhoGDIβ; however, we did not observe an inhibitory effect for the dominant-negative ZAK. Clearly, the kinase activities of ZAK are necessary for the negative regulation of RhoGDIβ functions, including G1 cell cycle arrest and hypertrophic growth.

The physical interaction of ZAK and RhoGDIβ results in subsequent inactivation of RhoGDIβ by phosphorylation, and might require docking interactions. The RhoGDIβ docking domain is localized to the last 100 C-terminal residues of ZAK. One possible hypothesis is that binding of ZAK to RhoGDIβ might change the localization and/or subcellular concentrations of both proteins as well as the biological functions of ZAK. Interference with the cellular activities of ZAK by RhoGDIβ, as evidenced by the results of the pull-down assay, measurements of cell size, and growth of the transfected cells, might be partially explained by maintenance of the bound ZAK in a hypophosphorylated form. Comparison of the cell size of ZAK-expressing cells with ZAK- and RhoGDIβ-expressing cells showed a relative decrease in cell size of 1.74- to 1.32-fold. We also found that ZAK kinase activities were decrease in cells with the co-expression of ZAK and RhoGDIβ (data not show). In addition, the fact that the growth rate of ZAK- and RhoGDIβ-expressing cells was similar to ZAKdn-expressing cells and faster than RhoGDIβ-expressing cells indicates that RhoGDIβ inhibits the biological functions of ZAK.

## Competing interests

The authors declare that they have no competing interests.

## Authors' contributions

CYH performed the pull-down assay. LCY performed the siRNA knockdown. KYL performed immunoprecipitation and detection of ZAK kinase activity. PHL performed the cell staining. JIYC was involved in measurement and growth. MYC and WWL performed Western blot analysis. JJY wrote the manuscript.

## Supplementary Material

Additional file 1 **Supplementary figure 1**. Binding of GST-RhoGDIb in a GST pull-down assay with ZAK-6-7. ZAK-100 (-100) is the positive control, and ZAK-200 (-200) is the negative control. Click here for file

Additional file 2 **Supplementary figure 2**. Series of different carboxyl terminal pEGFPc1-ZAK deletion mutants. Click here for file
