# Supplementary figures and images for: RhoGDIβ-induced hypertrophic growth in H9c2 cells is negatively regulated by ZAK
Source: J Biomed Sci. 2009 Jan 22;16(1):11. doi: 10.1186/1423-0127-16-11 (PMC2653512; doi:10.1186/1423-0127-16-11)

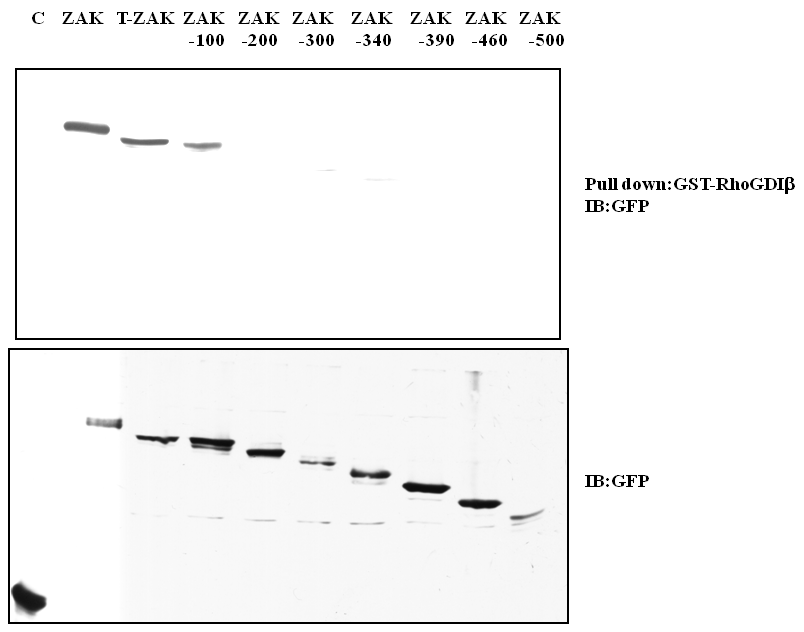

Supplement: Additional file 1 — Supplementary figure 1. Binding of GST-RhoGDIb in a GST pull-down assay with ZAK-6-7. ZAK-100 (-100) is the positive control, and ZAK-200 (-200) is the negative control. [file 1423-0127-16-11-S1.tiff]

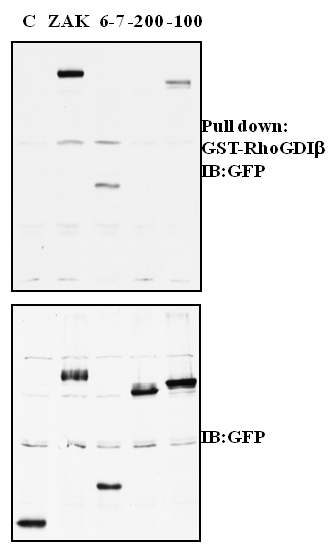

Supplement: Additional file 2 — Supplementary figure 2. Series of different carboxyl terminal pEGFPc1-ZAK deletion mutants. [file 1423-0127-16-11-S2.tiff]
